# Optimization and Predictive Modeling of Reinforced Concrete Circular Columns

**DOI:** 10.3390/ma15196624

**Published:** 2022-09-23

**Authors:** Gebrail Bekdaş, Celal Cakiroglu, Sanghun Kim, Zong Woo Geem

**Affiliations:** 1 Department of Civil Engineering, Istanbul University-Cerrahpasa, 34320 Istanbul, Turkey; 2 Department of Civil Engineering, Turkish-German University, 34820 Istanbul, Turkey; 3 Department of Civil and Environmental Engineering, Temple University, Philadelphia, PA 19122, USA; 4 Department of Smart City & Energy, Gachon University, Seongnam 13120, Korea

**Keywords:** predictive modeling, optimization, structural design

## Abstract

Metaheuristic optimization techniques are widely applied in the optimal design of structural members. This paper presents the application of the harmony search algorithm to the optimal dimensioning of reinforced concrete circular columns. For the objective of optimization, the total cost of steel and concrete associated with the construction process were selected. The selected variables of optimization include the diameter of the column, the total cross-sectional area of steel, the unit costs of steel and concrete used in the construction, the total length of the column, and applied axial force and the bending moment acting on the column. By using the minimum allowable dimensions as the constraints of optimization, 3125 different data samples were generated where each data sample is an optimal design configuration. Based on the generated dataset, the SHapley Additive exPlanations (SHAP) algorithm was applied in combination with ensemble learning predictive models to determine the impact of each design variable on the model predictions. The relationships between the design variables and the objective function were visualized using the design of experiments methodology. Applying state-of-the-art statistical accuracy measures such as the coefficient of determination, the predictive models were demonstrated to be highly accurate. The current study demonstrates a novel technique for generating large datasets for the development of data-driven machine learning models. This new methodology can enhance the availability of large datasets, thereby facilitating the application of high-performance machine learning predictive models for optimal structural design.

## 1. Introduction

**Figure 1:**
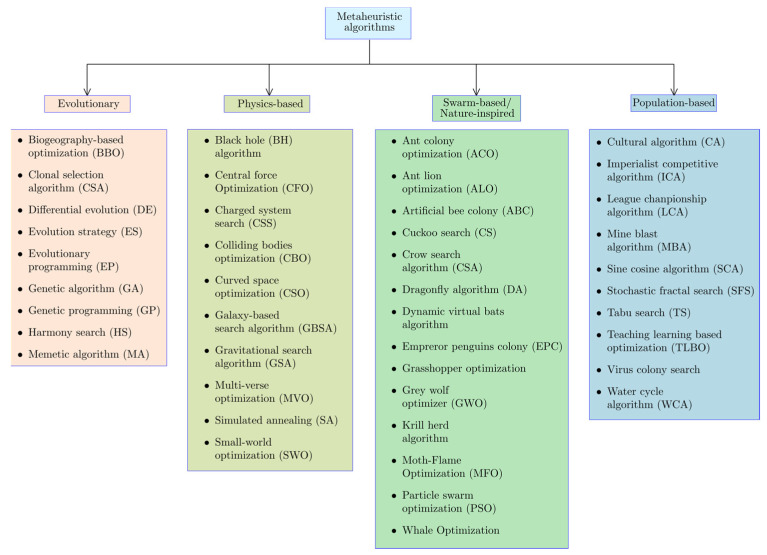
Classification of metaheuristic optimization algorithms.

Structural optimization aims at designing structures with the best possible dimensions that minimize cost without any impact on structural performance. In recent years metaheuristic optimization techniques have been increasingly applied to the optimization of different structures such as cylindrical reinforced concrete walls [1,2], retaining walls [3,4,5], plate girders [6], laminated composite plates [7,8,9], concrete-filled steel tubes [10,11], truss systems [12], timber structures [13], and liquid mass dampers [14,15,16]. These algorithms can be divided into evolutionary, physics-based, swarm-based, and population-based algorithms [17,18,19]. A detailed classification of the state-of-the-art metaheuristic optimization techniques can be found in Figure 1 [20,21,22,23,24,25,26,27,28,29,30,31,32,33,34,35]. Among the metaheuristic optimization algorithms applied to structural optimization, the harmony search algorithm stands out as one of the most widely used techniques. Besides structural optimization, the harmony search technique has been applied to various areas of science and engineering such as transportation engineering [36,37,38,39], environmental engineering [40,41], healthcare systems [42], bioinformatics [43,44,45], and cloud computing [46].

**Figure 2:**
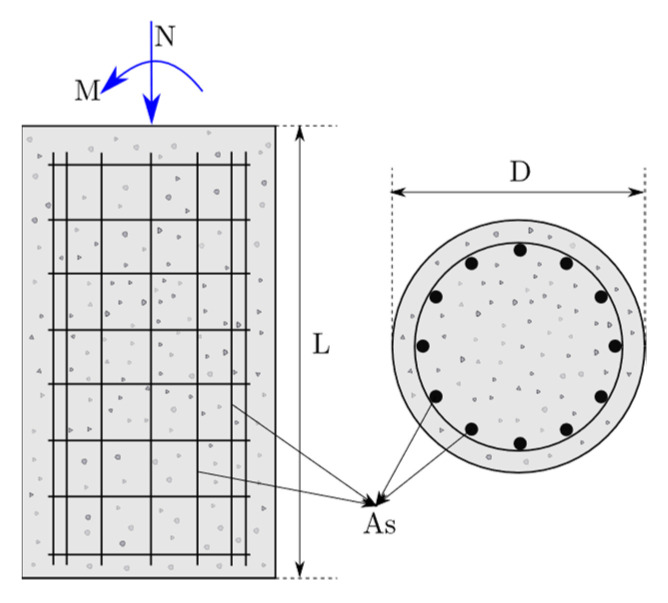
Cross-section and longitudinal section of a circular RC column.

Reinforced concrete (RC) circular columns have been used in broad applications in structural engineering. The total amount of longitudinal reinforcement determines their load-carrying capacity of them. Therefore, the accurate determination of the right amount of reinforcement in these structural members under axial forces and bending moments bears the utmost importance. Figure 2 shows a general description of an RC circular column where the lateral and longitudinal reinforcements can be seen. The outer diameter D and the total length L describe the geometry of the column in addition to the longitudinal reinforcement area $A_{s}$.

This paper presents a novel data-driven technique for the prediction of the area of the longitudinal reinforcement ($A_{s})\;$ in RC circular columns. To this end, four different ensemble learning algorithms have been utilized to obtain predictive models. The performances of these algorithms in terms of predicting $A_{s}$ accurately have been compared using the coefficient of determination (R^2^), mean absolute error (MAE), and root mean square error (RMSE) as the metrics of accuracy. The datasets needed to train these predictive models have been generated using the harmony search algorithm such that each data sample corresponds to an optimal design configuration that satisfies certain load-carrying requirements defined by the design codes. A combination of axial force and the bending moment was applied in each data sample. A dataset of 3125 samples was generated where each sample consists of six input variables and the corresponding output variable. The input variables in this dataset consist of the outer diameter of the column (D), the unit cost of concrete used in the construction ($C_{c}\;$), the unit cost of steel ($C_{s}$), the total length of the column (L), the bending moment acting on the column (M), and the axial force acting on the column (N). The corresponding output variable is the optimal longitudinal reinforcement area ($A_{s}$). These input variables were selected in order to have a description of the geometry, material properties, and external loading for each data sample. In this regard, the unit costs of concrete and steel quantify the material properties whereas the column length and outer diameter describe the geometry corresponding to a data sample. To clarify the impact of each input variable on the output of the predictive models and to show the dependencies between different variables, the SHAP algorithm has been utilized. Furthermore, a four-level factorial analysis has been carried out to visualize the variation of the output variable for the different levels of each input variable [47]. Based on the dependencies of the input variables, a predictive equation has been proposed for the reinforcement area. The equation has been developed using the harmony search algorithm to minimize the difference between the predicted and true optimal reinforcement areas. An R^2^ score of 0.998 could be achieved by the developed equation.

The current paper investigates the optimal design of circular RC columns under combined loading, which is an area of structural engineering that has not been investigated using data-driven machine learning techniques to the best of the authors’ knowledge. The work related to failure mode classification and capacity prediction of RC columns using an ensemble machine learning algorithm AdaBoost by Feng et al. [48] can be counted among the recent machine learning-related research works in the field of conventional RC columns. The ensemble learning algorithm was developed based on a data set consisting of 254 data samples collected from cyclic loading tests. Also, Dogan et al. [49] investigated the damage levels of RC columns under cyclic lateral loading conditions using machine learning methods for classification such as support vector machines, K-nearest neighbors and discriminant analysis. The machine learning models were trained on a set of 390 damage images. However, the research activity in the area of RC columns using machine learning methods has been limited compared to other areas such as concrete columns with fiber-reinforced polymer wrappings [50,51], or crack propagation and corrosion in RC structures [52,53,54]. Nasrollahzadeh and Nouhi [50] proposed fuzzy inference system models to predict the strength and strain capacity of square concrete columns wrapped with fiber-reinforced polymer. Experimental data sets consisting of 261 and 112 test samples were used for the prediction of compressive strength and ultimate strain respectively. Naderpour et al. [51] utilized artificial neural network and gene expression programming techniques to predict the compressive strength of columns confined with fiber reinforced polymers based on a data set consisting of 95 data samples. However, despite the benefits of using composite materials as reinforcement or confinement for columns reported in the literature, the overwhelming majority of new constructions and existing infrastructure rely on conventional reinforced concrete.

One of the reasons for the lack of machine learning related research in the field of RC columns is the difficulty of obtaining large datasets. Machine learning-based predictive models need to be trained using large and comprehensive data sets in order to be relevant in general-purpose structural design. On the other hand, experimental research in the field of RC members is generally costly and experimental programs usually deliver a limited number of data points. Therefore, alternative techniques need to be devised for the training of machine learning models in order to use these powerful techniques in the field of RC design.

An important current issue in previous literature pertaining to machine learning applications in structural engineering is the size of the data sets used during the model training process. Evidently, most studies in this field depend on data sets with less than a thousand data samples. However, the reliability of a machine learning model heavily depends on the size and quality of the data used in its training. The aim of the current study is to present a methodology for the generation of large datasets related to the optimal design of RC columns. The novelty of this current work shows the applicability of newly developed techniques of artificial intelligence to the design process of RC columns. The availability of quality data is a major requirement in this process. However, large datasets are needed for the development of accurate predictive machine learning models, which is one of the limiting factors in the process of machine learning model development. The current paper proposes a novel technique for the generation of large datasets. This technique is highly valuable since it can remove one of the major bottleneck points in the application of machine learning techniques to structural design.

## 2. Dataset Generation and Analysis

A large dataset consisting of 3125 unique configurations has been generated with the help of the harmony search optimization algorithm. Each sample in this dataset corresponds to a design configuration that minimizes the total cost associated with the construction process while keeping the load-carrying capacity above a certain level. To this end, the axial load and moment capacities were kept above the applied load which can be described in Equation (1):

(1) $$\phi P_{n}\geq P_{u},\phi M_{n}\geq M_{u}$$

In Equation (1), $P_{u}$ and $M_{u}$ are the applied loads, $P_{n}$ and $M_{n}$ are the nominal strengths of the column cross-section, and ϕ is the strength reduction factor. This process starts with the generation of a randomly populated harmony memory matrix (HM) as shown in Equation (2) where f denotes the cost function, HMS is the size of the solution candidate population, $V_{c}$ and $W_{s}$ are the total volume of concrete and the total weight of steel respectively and x^i^ is a solution candidate vector containing the variables $D^{i}$, $C_{c}^{i}$, $C_{s}^{i}$, $L^{i}$, $M^{i}$, $N^{i}$, $A_{s}^{i}$. The cost function f determines the performance of each population member and the solution candidates can be ranked accordingly.

(2) $$\begin{array}{c} \mathrm{HM}=\left[\begin{array}{ccc} \begin{array}{c} D^{1}\\ \begin{array}{c} D^{2}\\ \vdots \\ D^{\mathrm{HMS}} \end{array} \end{array} & \begin{array}{c} C_{c}^{1}\\ \begin{array}{c} C_{c}^{2}\\ \vdots \\ C_{c}^{\mathrm{HMS}} \end{array} \end{array} & \begin{array}{ccc} \begin{array}{c} C_{s}^{1}\\ \begin{array}{c} C_{s}^{2}\\ \vdots \\ C_{s}^{\mathrm{HMS}} \end{array} \end{array} & \begin{array}{c} L^{1}\\ \begin{array}{c} L^{2}\\ \vdots \\ L^{\mathrm{HMS}} \end{array} \end{array} & \begin{array}{ccc} \begin{array}{c} M^{1}\\ \begin{array}{c} M^{2}\\ \vdots \\ M^{\mathrm{HMS}} \end{array} \end{array} & \begin{array}{c} N^{1}\\ \begin{array}{c} N^{2}\\ \vdots \\ N^{\mathrm{HMS}} \end{array} \end{array} & \begin{array}{cc} \begin{array}{c} A_{s}^{1}\\ \begin{array}{c} A_{s}^{2}\\ \vdots \\ A_{s}^{\mathrm{HMS}} \end{array} \end{array} & \begin{array}{c} \begin{array}{c} \begin{array}{c} \begin{array}{c} f\left(x^{1}\right)\\ \begin{array}{c} f\left(x^{2}\right)\\ \vdots \\ f\left(x^{\mathrm{HMS}}\right) \end{array} \end{array} \end{array} \end{array} \end{array} \end{array} \end{array} \end{array} \end{array}\right]\\ f\left(x^{i}\right)={\left(C_{c}\right)}^{i}V_{c}+{\left(C_{s}\right)}^{i}W_{s} \end{array}$$

In Equation (2), $C_{c}^{i}$, $C_{s}^{i}$, $L^{i}$, $M^{i}$, $N^{i}$, and $A_{s}^{i}$ stand for the concrete cost per unit volume, steel cost per unit weight, length of the column, bending moment, axial force and the total area of the longitudinal reinforcement in the i-th solution candidate respectively. The harmony search technique obtains an optimum solution that minimizes the total cost by an evolutionary process in which the solution candidates improve incrementally and eventually converge to an optimum solution. The evolutionary process of incremental improvement of the harmony memory matrix can be described in Equations (3)–(6).

(3) k=int(rand⋅HMS), rand∈(0,1)

(4) $$x_{i,\mathrm{new}}=x_{i,\;\min}+\mathrm{rand}\cdot \left(x_{i,\;\max}-x_{i,\;\min}\right),\;\mathrm{if}\;\mathrm{HMCR}\;+\;\mathrm{rand}$$

(5) $$x_{i,\mathrm{new}}=x_{i,k}+\mathrm{rand}\cdot \mathrm{PAR}\cdot \left(x_{i,\;\max}-x_{i,\;\min}\right),\;\mathrm{if}\;\mathrm{HMCR}\;+\;\mathrm{rand}$$

(6) $$\mathrm{HMCR}=0.5\left(1-\frac{i}{\max\left(i\right)}\right),\;\mathrm{PAR}=0.05\left(1-\frac{i}{\max\left(i\right)}\right)$$

In Equations (3)–(6), HMCR, PAR, $x_{i,\;\min}$ and $x_{i,\;\max}$ stand for the harmony memory consideration rate, the pitch adjustment rate, the minimum and the maximum values of the i-th input variable in the population respectively. After each harmony search iteration, the updated solution candidates replace the old ones if they have superior performance and satisfy the structural design code requirements. For a more detailed review of the harmony search technique, the reader is referred to [55].

**Figure 3:**
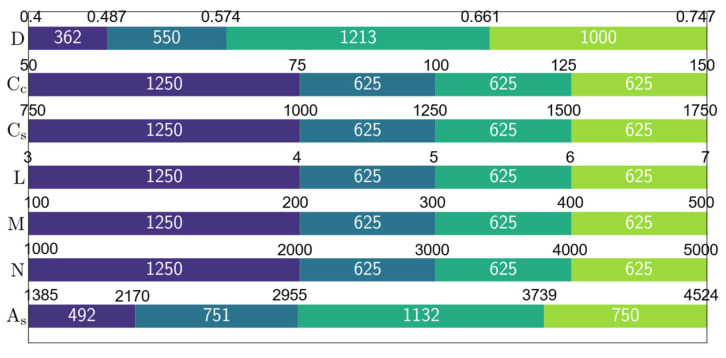
Design variable ranges in the dataset.

The variable ranges of the dataset generated using the harmony search algorithm can be seen in Figure 3 and Table 1. In Figure 3, the variable ranges have been divided into four partitions and the ranges of these partitions have been shown on the horizontal bars whereas the number of samples in each partition is shown inside the horizontal bars. The length of each partition in Figure 3 is in proportion to the number of samples belonging to that partition. Figure 3 shows that the largest partition for the outer diameter D consists of 1213 samples ranging between 0.574 m and 0.661 m. The second largest partition for this variable with 1000 samples ranges between 0.661 m and 0.747 m. The remaining two partitions ranging between the lower bound of the outer diameter of 0.4 m and 0.574 m constitute 29% of the entire dataset. The partitions for the variables $C_{c}$, $C_{s}$, L, M, and N are evenly distributed. The largest partition for the column length L consists of 1250 samples ranging between 3 m and 4 m, which is 40% of the entire dataset. The horizontal bars for $C_{c}\;\;$ and $C_{s}$ in Figure 3 show the unit prices of concrete and steel in USD/m^3^ and USD/ton respectively.

**Figure 4:**
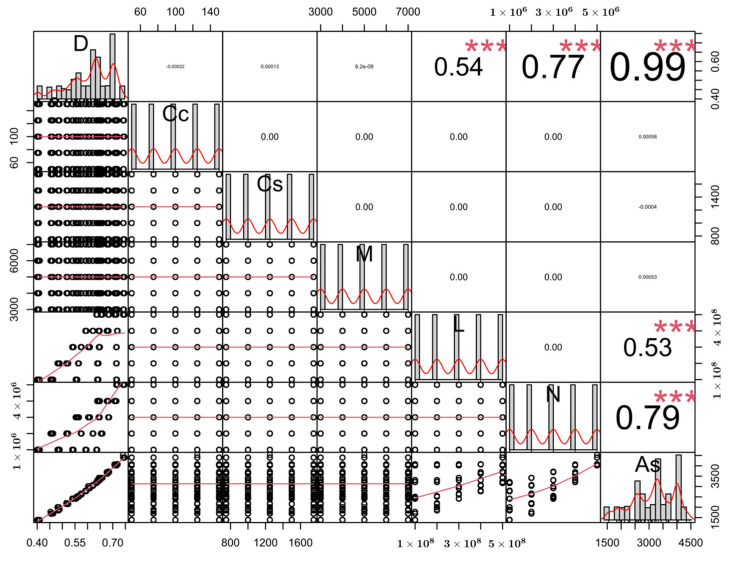
Correlation matrix of the dataset.

Table 1 includes the upper and lower bounds as well as statistical properties of the design variables represented in the dataset. These statistical properties are the average value, standard deviation, and variance of each variable inside the dataset. Furthermore, for each variable, the corresponding boundaries and statistical properties have been listed after normalizing the variables by their average values. These normalized values are used at a later stage for the development of a predictive equation. Also, the partitions presented in Figure 3 are used as the basis of a four-level factorial analysis to determine the variation of $A_{s}$ for each design variable. In addition to the partition plot in Figure 3, also a correlation plot has been generated for the dataset (Figure 4). For each input variable and the output variable $A_{s}$_,_ Figure 4 shows the Pearson correlation coefficient between any two of these variables in the upper right portion of the diagram. Pearson correlation values close to 1 indicate a high correlation between two variables. The highest correlation coefficient in Figure 4 can be observed between $A_{s}$ and D which indicates that the outer diameter has a significant impact on the reinforcement area. The second highest correlation can be observed between $A_{s}$ and N with a Pearson correlation value of 0.79. Another relatively high correlation is observed between D and N with a correlation value of 0.77. Finally, the column length L is correlated to $A_{s}$ and D with correlation coefficients of 0.53 and 0.54 respectively. Greater correlation between variables is represented by larger font size and stars inside the tiles of the matrix. In Figure 4, each variable occupies one of the diagonal tiles and the scale of this variable is shown in one of the horizontal axes and one of the vertical axes. Furthermore, each diagonal tile contains a histogram showing the distribution of the variable in it. The lower left portion of the correlation matrix contains bivariate scatter plots with regression lines. The equation for the computation of the Pearson correlation coefficient as well as the other metrics of accuracy used in this paper can be found in Appendix A.

**Figure 5:**
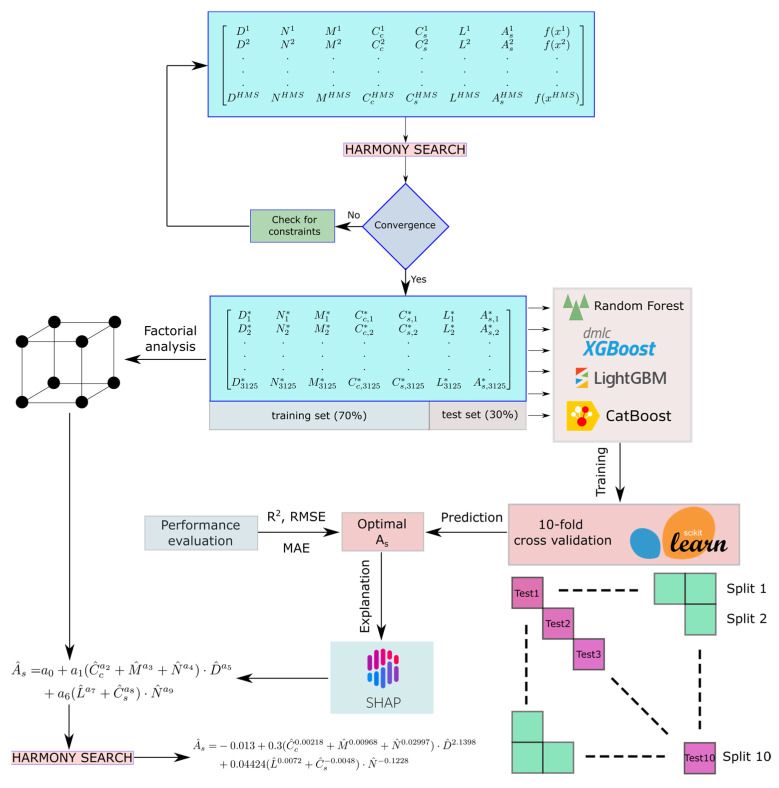
Machine learning and equation development process.

Using the dataset whose properties have been shown in Figure 3, Table 1, and Figure 4, four different data-driven predictive models have been trained using the ensemble learning algorithms XGBoost, LightGBM, Random Forest, and CatBoost. The results of them and their interpretations using the SHapley Additive exPlanations (SHAP) technique have been presented in the next section. The theoretical background of ensemble learning and SHAP algorithms can be found in [3,56,57,58,59,60,61,62]. In addition to the SHAP analysis also, a four-level factorial analysis has been carried out to further investigate the sensitivity of $A_{s}$ to the variations in different design variables. Afterward, a closed-form equation has been proposed for the prediction of $A_{s}$ as a function of the six design variables shown in Figure 3 and Figure 4. The overall process of dataset generation, training of the machine learning models, and the development of a predictive closed-form equation have been summarized in a flow chart in Figure 5.

## 3. Results

This section presented the comparison of the optimal reinforcement areas predicted by the ensemble learning algorithms with the true optimal values obtained through the harmony search algorithm. The performances of each predictive model have been measured by the metrics of the coefficient of determination, mean absolute error and root mean squared error. The outcome of the ensemble learning models has been analyzed with the SHAP algorithm to determine the variables with the highest impact on the model predictions. Furthermore, a four-level factorial analysis has been performed to visualize the variation of the optimal reinforcement cross-section for each input variable. Based on the outcome of the SHAP and factorial analyses, a predictive equation format has been proposed. This predictive equation has been developed using the harmony search algorithm, and the accuracy of the obtained equation has been demonstrated by the same accuracy metrics applied to the ensemble learning models.

### 3.1. Ensemble Learning Model Predictions

**Figure 6:**
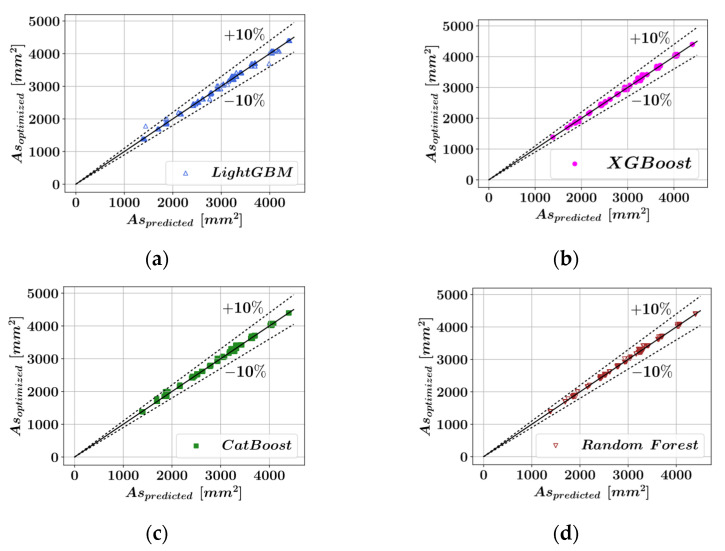
Comparison of the predicted and optimized dimensions. (**a**) LightGBM. (**b**) XGBoost. (**c**) CatBoost. (**d**) Random Forest.

The ensemble learning models have been trained by splitting the entire dataset into a training set and a test set in 70% to 30% proportions. This division was made based on past machine learning studies in the area of structural engineering. Particularly, the study of Nguyen et al. [63] demonstrated that among the 10/90, 20/80, 30/70, 40/60, 50/50, 60/40, 70/30, 80/20, and 90/10 training set to test set ratios, the 70/30 ratio delivered the best performance. The models have been trained on the training set using ten-fold cross-validation. After the completion of the model training, the test set was used to measure the model performances. The performances of the ensemble learning models have been visualized by plotting the true optimal $A_{s}$ values of the test set against the $A_{s}$ values predicted by the ensemble learning models. Figure 6 shows the comparison of the predicted and actual optimal $A_{s}$ values for each of the four predictive models. In Figure 6, the diagonal solid lines represent the case when the actual and predicted values are equal, whereas the dotted lines represent ± 10% deviation from a perfect prediction. The performances of these predictive models are compared to each other in Table 2. According to Table 2, the Random Forest algorithm demonstrated the best performance in terms of both prediction accuracy on the test set and the speed of execution (3.71 s).

### 3.2. SHAP Analysis

**Figure 7:**
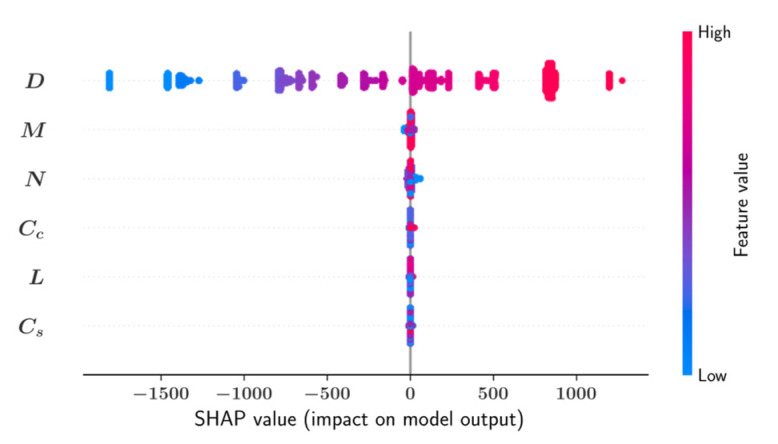
SHAP summary plot for $A_{s}$ (Random Forest).

The SHAP analysis visually describes the contribution of each design variable to the prediction of a machine learning model. The SHAP summary plot and feature dependence plots in this section are based on the Random Forest algorithm selected due to its superior performance on this dataset. The SHAP summary plot in Figure 7 ranks the six input variables according to their impact on the predictive model output. In Figure 7, each data sample is represented by a dot and positive SHAP values indicate an increasing effect of a variable on the model output whereas negative SHAP values indicate a decreasing effect on the model output. The impact of a variable on model output for a particular data sample is a function of the position of a dot along the horizontal axis. On the other hand, the numerical values of the input parameters are represented with color so that high parameter values are shown with shades of red and the low parameter values are shown with shades of blue. According to Figure 7, the outer diameter D has the greatest impact on the model output. It can be deduced that an increase in the value of D also leads to an increase in the model prediction. Conversely, decreasing the D value leads to lower model predictions. On the other hand, the impacts of the remaining parameters on the model output are an order of magnitude smaller than the impact of D according to Figure 7.

**Figure 8:**
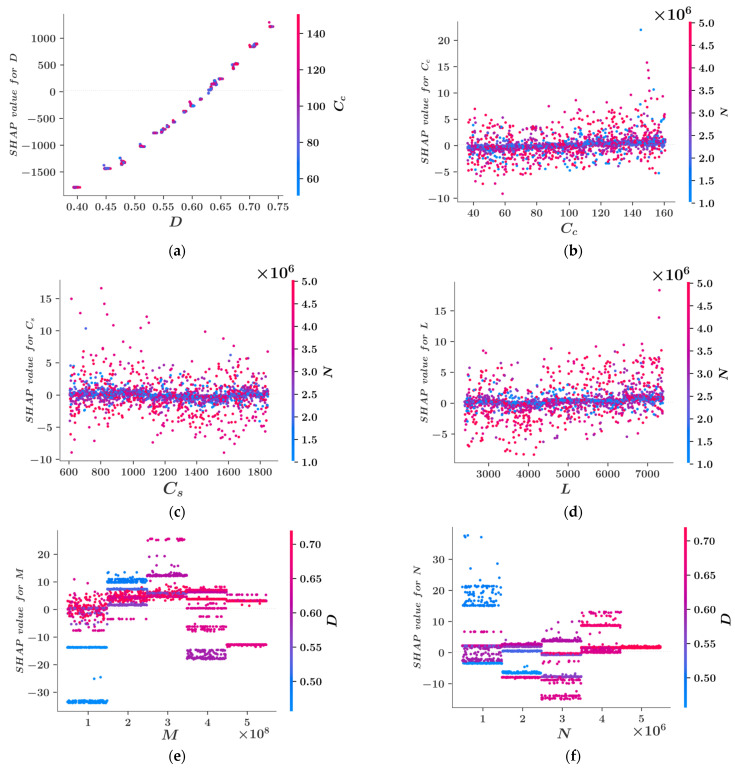
Feature dependence plots for the variables (**a**) D, (**b**) $C_{c}$, (**c**) $C_{s}$, (**d**) L, (**e**) M, (**f**) N.

The feature dependence plots in Figure 8 can help better understand the interdependencies between different variables and their effects on the model output. Figure 8a clearly shows that as the D value increases, so does the SHAP value for this variable. This confirms the inference from Figure 7 that increased D values lead to increased reinforcement area. The colors of the dots in Figure 8a indicate the numerical values of $C_{c}$ which is the variable most dependent on D. On the other hand, the feature dependence plots of $C_{c}$, $C_{s}$ and L show that the SHAP values of these variables have a horizontal trend as the variable values increase. For these three variables, most of the SHAP values stay in the range of −5 to 5 for the entire dataset. For a significant portion of the dataset, the SHAP values are clustered around zero. Also, for all three of these variables, N is the variable most dependent on them. The feature dependence plots of M and N exhibit certain patterns for different levels of these variables. Figure 8e,f show that the feature dependence plots for M and N are fragmented and can be investigated separately for different levels of these variables. For each value of M and N, the SHAP values of these variables are concentrated around different levels depending on the value of D which is the parameter most dependent on M and N. It can be observed that M has the greatest impact on the model output when $M=3\times 10^{8}\mathrm{Nmm}$ and D have large values shown in red and the least impthe act when $M=10^{8}\mathrm{Nmm}$ and D have small values shown in blue. Similarly, N has the greatest impact when $N=10^{6}N$ and D have small values shown in blue and the least impact when $N=3\times 10^{6}N$ and D have large values shown in red.

### 3.3. Four-Level Factorial Analysis

**Figure 9:**
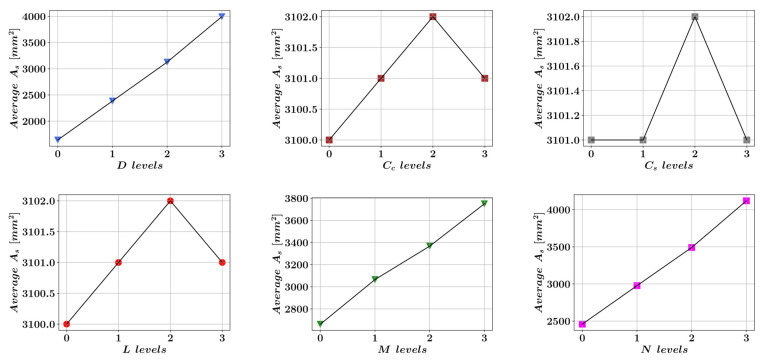
Four-level factorial analysis.

The factorial analysis technique is widely used for gaining insights into the response and sensitivity of a system that depends on multiple variables to the variations in a single variable. The factorial analysis technique is particularly useful when the variables of a system can be broken down into different levels. In this paper, the cross-section of reinforcement is predicted as a function of six variables. Each of these variables has been broken down into four levels as shown in Figure 3. The four-level factorial analysis enables the visualization of the nonlinear variations in the target variables or curvatures in the system response [64]. Afterward, for each level of each variable, the average value of the area of reinforcement has been calculated. These average values are plotted for different levels of each variable in Figure 9. A significant variation of $A_{s}$ in Figure 9 with respect to changes in a certain variable indicates the high sensitivity of $A_{s}$ to the changes in this variable. According to Figure 9, the greatest change in the value of $A_{s}$ is observed when D increases from its lowest level (level 0) to its highest level (level 3). A total increase from 1641 mm^2^ to 3993 mm^2^ can be observed which corresponds to a 143% increase. The second largest increase in the average value of $A_{s}$ can be observed when N increases from its lowest level to its highest level. In this case, the increase of the area from 2459 mm^2^ to 4119 mm^2^ can be observed which corresponds to a 68% increase. The third largest percentage-wise increase in the average $A_{s}$ value can be observed when M increases from its lowest level to its highest level. An increase from an average area of 2661 mm^2^ to an average area of 3750 mm^2^ can be observed which corresponds to a 41% increase. For the remaining three variables $C_{c}$, $C_{s}$ and L, the changes in the average value of $A_{s}$ was negligible in comparison to D, N, and M.

### 3.4. Development of an Equation for the Prediction of the Optimum Reinforcement Area

In light of the results presented in the previous sections, the formula in Equation (7) has been proposed for the prediction of the reinforcement area in an optimal design. In Equation (7) the variables ${\hat{A}}_{s},{\hat{C}}_{c},\hat{M},\hat{N},\hat{D},\hat{L},{\hat{C}}_{s}$ are normalized by the average value of each variable in the dataset consisting of 3125 samples.

(7) $${\hat{A}}_{s}=a_{0}+a_{1}\left({\hat{C}}_{c}^{a_{2}}+{\hat{M}}^{a_{3}}+{\hat{N}}^{a_{4}}\right)\cdot {\hat{D}}^{a_{5}}+a_{6}\left({\hat{L}}^{a_{7}}+{\hat{C}}_{s}^{a_{8}}\right)\cdot {\hat{N}}^{a_{9}}$$

The coefficients $a_{0}$ to $a_{9}$ in Equation (7) have been adjusted using harmony search iterations. This process necessitates the declaration of a new harmony memory matrix that contains the coefficients of Equation (7) as shown in Equation (8) where the population consists of 30 different solution candidates.

(8) $$\mathrm{HM}=\left[\begin{array}{ccc} \begin{array}{c} a_{0}^{1}\\ \begin{array}{c} a_{0}^{2}\\ \vdots \\ a_{0}^{30} \end{array} \end{array} & \begin{array}{c} a_{1}^{1}\\ \begin{array}{c} a_{1}^{2}\\ \vdots \\ a_{1}^{30} \end{array} \end{array} & \begin{array}{ccc} \begin{array}{c} a_{2}^{1}\\ \begin{array}{c} a_{2}^{2}\\ \vdots \\ a_{2}^{30} \end{array} \end{array} & \begin{array}{c} a_{3}^{1}\\ \begin{array}{c} a_{3}^{2}\\ \vdots \\ a_{3}^{30} \end{array} \end{array} & \begin{array}{ccc} \begin{array}{c} a_{4}^{1}\\ \begin{array}{c} a_{4}^{2}\\ \vdots \\ a_{4}^{30} \end{array} \end{array} & \begin{array}{c} a_{5}^{1}\\ \begin{array}{c} a_{5}^{2}\\ \vdots \\ a_{5}^{30} \end{array} \end{array} & \begin{array}{ccc} \begin{array}{c} a_{6}^{1}\\ \begin{array}{c} a_{6}^{2}\\ \vdots \\ a_{6}^{30} \end{array} \end{array} & \begin{array}{c} a_{7}^{1}\\ \begin{array}{c} a_{7}^{2}\\ \vdots \\ a_{7}^{30} \end{array} \end{array} & \begin{array}{cc} \begin{array}{c} a_{8}^{1}\\ \begin{array}{c} a_{8}^{2}\\ \vdots \\ a_{8}^{30} \end{array} \end{array} & \begin{array}{c} a_{9}^{1}\\ \begin{array}{c} a_{9}^{2}\\ \begin{array}{c} \vdots \\ a_{9}^{30} \end{array} \end{array} \end{array} \end{array} \end{array} \end{array} \end{array} \end{array}\right]$$

**Figure 10:**
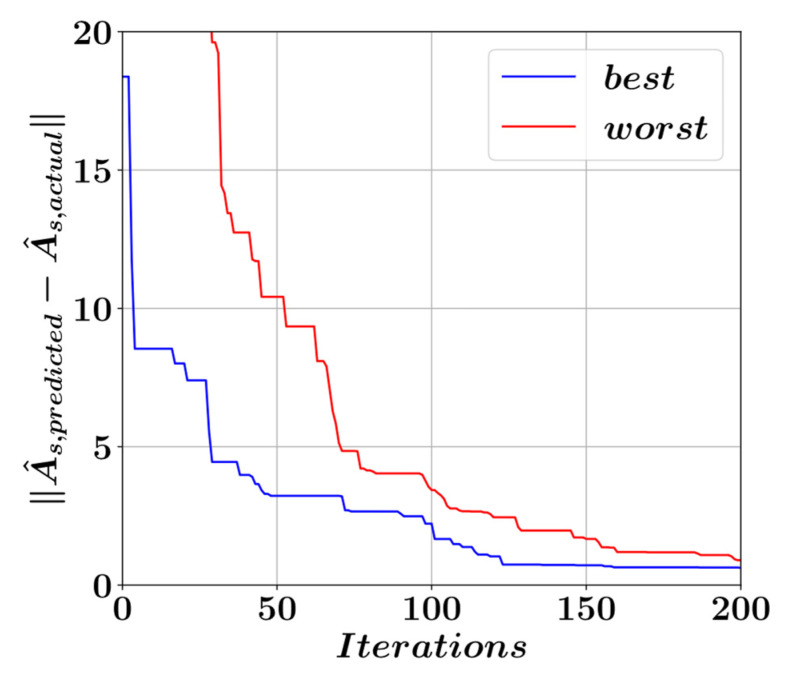
Equation performance throughout the harmony search iterations.

After every harmony search iteration, the performances of the solution candidates are measured by comparing their predictions of ${\hat{A}}_{s}$ with the actual ${\hat{A}}_{s}$ values for the entire dataset. The prediction error is represented by the Euclidean norm of the vector containing the differences between the actual and predicted optimal ${\hat{A}}_{s}$ values. The development of these vector norms for the best- and worst-performing members of the harmony memory population is presented in Figure 10.

**Figure 11:**
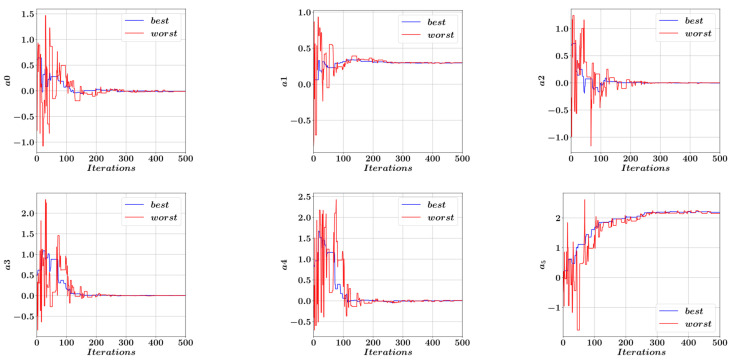

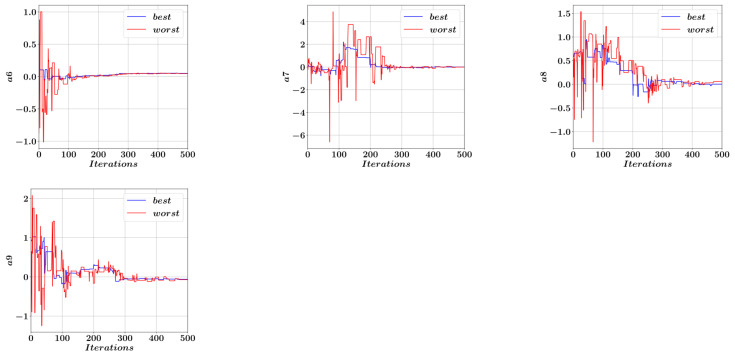
Development of the equation coefficients in Equation (7).

A total of 5000 harmony search iterations was carried out to obtain the best possible equation coefficients with the smallest possible error norm. Figure 10 shows the development of the best and worst solution candidates in the initial 200 iterations. It should be noted that the largest improvements in the solution candidates take place during the initial phases of the harmony search iterations. Figure 11 shows the process of obtaining the coefficients $a_{0}$ to $a_{9}$ that minimize the difference between the optimal ${\hat{A}}_{s}$ values predicted by Equation (7) and the actual optimal ${\hat{A}}_{s}$ values.

Figure 11 shows the values of the coefficients $a_{0}$ to $a_{9}$ in the first 500 harmony search iterations. The coefficient values corresponding to the best- and worst-performing members of the harmony memory population are shown in blue and red respectively. It can be observed that after the initial fluctuations, these coefficients tend to converge to their optimal limit values. Inserting these limit values of the coefficients $a_{0}$ to $a_{9}$ in Equation (7), we obtain Equation (9) for the prediction of the normalized reinforcement area ${\hat{A}}_{s}$ from which the actual reinforcement area $A_{s}$ can be obtained after multiplication with the average value of $A_{s}$ from Table 1.

(9) $$\begin{array}{c} {\hat{A}}_{s}=-0.013+0.3\left({\hat{C}}_{c}^{0.00218}+{\hat{M}}^{0.00968}+{\hat{N}}^{0.02997}\right)\cdot {\hat{D}}^{2.1398}\\ \;+\;0.04424\left({\hat{L}}^{0.0072}+{\hat{C}}_{s}^{-0.0048}\right)\cdot {\hat{N}}^{-0.1228} \end{array}$$

**Figure 12:**
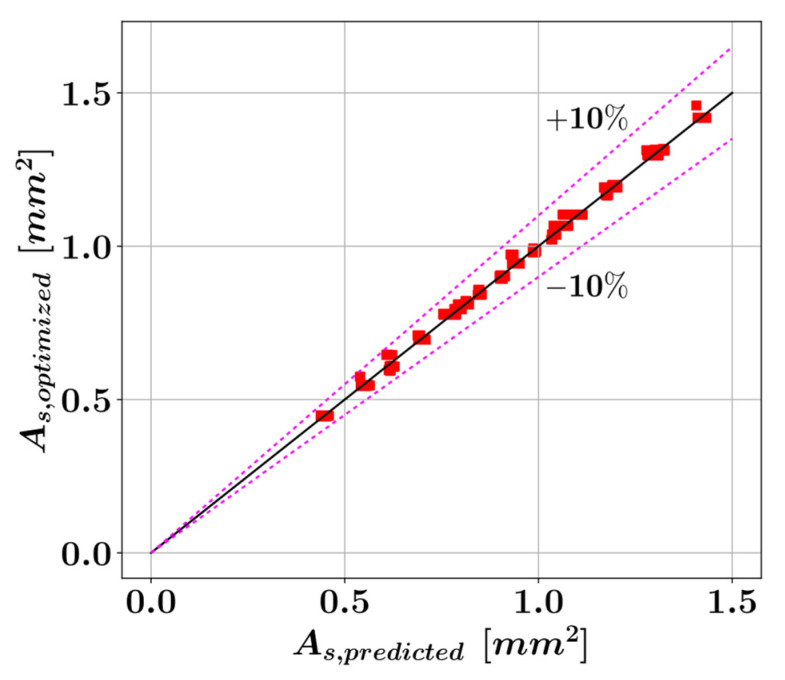
Comparison of the predicted and actual optimal reinforcement areas.

A coefficient of determination of 0.9985, mean absolute error of 0.0076, and root mean square error of 0.0099 could be achieved using Equation (9). The ${\hat{A}}_{s}$ values predicted by Equation (9) are plotted against the actual ${\hat{A}}_{s}$ values in Figure 12 where the dotted lines indicate the ±10% deviation from a perfect match between the predicted and actual values.

## 4. Discussion

Circular RC columns are ubiquitous in the structural design of buildings, bridges, and ports. Therefore, the optimal design of these structural members can have enormous economic and environmental benefits. This paper demonstrates the applicability of machine learning models to the structural optimization of RC circular columns. The total cross-sectional area of the longitudinal reinforcement was selected as the decisive parameter in the design of these structures. A novel technique has been applied in the training phase of these machine learning models. The harmony search algorithm was used to generate a large dataset consisting of 3125 samples where each sample represents an optimum configuration of column geometry and external loading. XGBoost, LightGBM, Random Forest, and CatBoost algorithms are used to generate four different ensemble learning predictive models. The performances of these models have been compared using the coefficient of determination (R^2^), mean absolute error (MAE) and root mean squared error (RMSE) as the metrics of accuracy. It was found that the Random Forest algorithm performed better than the other three ensemble learning algorithms in terms of both the accuracy of predicted optimal cross-sectional areas and the computational speed. However, it should be noted that all ensemble learning algorithms demonstrated high accuracy with an R^2^ score greater than 0.99. On the other hand, the execution speed of the CatBoost algorithm was significantly slower than the remaining three algorithms. The output of the Random Forest algorithm has been further analyzed using the SHAP algorithm to better understand the impact of each input variable on the model prediction and the interdependencies between the input variables. Furthermore, a four-level factorial analysis has been carried out to visualize the sensitivity of the reinforcement cross-sectional area (A_s_) to various input parameters.

The results of the four-level factorial analysis showed that the column diameter and applied external loads largely determine the cross-sectional area of steel reinforcement necessary for a safe design. The outer diameter of the column was also singled out by the SHAP summary plot as the most impactful design variable that determines the output of the random forest model. The outcome of the SHAP analysis and the factorial analysis can be interpreted as the external loading and column size being an order of magnitude more significant than the unit costs of steel and concrete in terms of their effect on the necessary amount of reinforcement.

In the design of a predictive equation, both the factorial analysis and the SHAP feature dependence plots have been decisive. In addition to a bias term, the predictive equation consists of two other terms. The first term after the bias term is chosen to be a product of the normalized diameter with the sum of the three parameters most dependent on D according to the feature dependence plots. The second term is chosen to be a product of the axial load with the sum of the normalized values of the two design variables most dependent on the axial load. In order to capture the nonlinear variations in the optimum reinforcement area, each term in the equation was raised to a power and multiplied by a coefficient. Afterward, the optimal coefficient values were determined through harmony search optimization. A coefficient of determination greater than 0.99 could be obtained by the predictive equation thus developed. However, it should be noted that more comprehensive studies including a larger number of design variables and greater ranges for the variable values should be performed to enhance the reliability of the developed equation.

Since machine learning techniques are data-driven, the applicability of these techniques to structural design depends on the availability of quality datasets. Furthermore, the accuracy of the obtained predictive models depends largely on the size of the dataset. However, most of the recent machine learning related research in the field of structural engineering is based on data sets not large enough to be statistically significant. This issue is being addressed in this study by proposing a harmony search-based novel technique for generating large data sets. The application of the harmony search methodology to the problem of data generation can solve the problem of limited data availability in structural engineering. Using optimization techniques such as harmony search, large datasets can be generated where the structural performances of the generated optimal samples are controlled according to the requirements in the existing design codes.

## 5. Conclusions

The performance of any predictive model depends mostly on the size and quality of the dataset used in its training. The current paper demonstrated a novel technique for the generation of large datasets using the harmony search optimization methodology. The generated dataset was used in the prediction of the optimal amount of longitudinal reinforcement for circular RC columns. Four different ensemble learning models were demonstrated to perform well in the prediction process. Furthermore, a closed-form equation was proposed that predicts the optimal amount of reinforcement that minimizes the cost associated with the construction process without violating the design code requirements. The most important results of this research work can be listed as follows:

- Four different machine learning models were developed using the XGBoost, LightGBM, Random Forest, and CatBoost algorithms. All of these algorithms performed well on the dataset with an R^2^ score greater than 0.99. Among these models, the Random Forest algorithm performed best in terms of both accuracy and computational speed whereas the CatBoost algorithm was nearly an order of magnitude slower than the rest of the algorithms.
- The results of the SHAP analysis showed that the outer diameter of the circular column has the greatest impact on the machine learning model predictions. The impacts of the applied axial loading (N) and bending moment (M) were found to be dependent on the value of D. At smaller values of D, N was shown to have a larger impact on the model output.
- After dividing the dataset into four segments for each variable the four-level factorial analysis showed that a 59% increase in the outer diameter can lead to a 143% increase in the optimal value of $A_{s}$. $A_{s}$ was also found to be highly sensitive to variations in N and M. Doubling the magnitude of N was observed to cause a 68% increase in the optimal value of $A_{s}$ whereas doubling the magnitude of M led to a 41% increase in the optimal value of $A_{s}$.
- A closed-form equation with an R^2^ score of 0.9985 was proposed which predicts the optimal value for $A_{s}$ as a function of column outer diameter, axial loading, bending moment, column length, and the unit prices of concrete and steel.

The availability of closed-form equations that deliver optimal dimensions for structural design can greatly facilitate and accelerate the design process for practicing engineers. With the help of these equations, the most favorable design combinations can be obtained without the need for complex optimization methodologies. However, it should be noted that the proposed equation and machine learning models in this paper are limited by the range of variables that constitute the dataset. Therefore, further research needs to be carried out for the development of more comprehensive predictive models. Furthermore, the scope of the variables included in the dataset could be enhanced to include variables such as the number and diameter of longitudinal reinforcing bars. In its current form, the output of the predictive equation could be evenly distributed to determine the area and a total number of the longitudinal reinforcing bars. Also, the spacing and dimensions of the lateral reinforcements can be included in the database. Future research towards the design of RC columns using machine learning methodologies can include composite materials such as carbon fiber or glass fiber reinforced polymers as the material of reinforcement.

## Figures and Tables

**Table 1:** Variable ranges and statistical properties.

Variable	Min		Max		Average		Standard Deviation		Variance	
	Actual	Normalized	Actual	Normalized	Actual	Normalized	Actual	Normalized	Actual	Normalized
D [m]	0.4	0.647	0.747	1.209	0.618	1	0.087	0.141	0.0076	0.02
Cc [USD/m^3^]	50	0.5	150	1.5	100	1	35.4	0.354	1250	0.125
Cs [USD/ton]	750	0.6	1750	1.4	1250	1	354	0.283	125,000	0.08
L [m]	3	0.6	7	1.4	5	1	1.41	0.283	2	0.08
M [kNm]	100	0.333	500	1.667	300	1	141	0.471	20,000	0.222
N [kN]	1000	0.333	5000	1.667	3000	1	1414	0.471	2,000,000	0.222
As [mm^2^]	1385	0.447	4524	1.459	3101	1	799	0.258	639,080	0.067

**Table 2:** Prediction accuracy of the machine learning models.

Algorithm	Variable	R^2^		MAE		RMSE		Duration [s]
		Train	Test	Train	Test	Train	Test	
XGBoost	As	0.9999	0.9995	1.998	7.523	3.839	17.072	5.14
Random Forest	As	0.9999	0.9996	2.593	7.111	6.095	15.929	3.71
LightGBM	As	0.9994	0.9988	9.962	12.767	19.673	27.157	6.07
CatBoost	As	0.9998	0.9994	7.579	10.788	12.440	18.940	28.23

## Data Availability

Data will be made available upon reasonable request.

## Appendix A

In Table A1, $x_{i}$ and $y_{i}$ denote the values of two different data series, ${\overset{\text{˜}}{y}}_{i}$ denotes the predicted values of the data series $y_{i}$, and n is the total number of data points in the series.

**Table A1:** Metrics of Model Accuracy.

Root mean square error (RMSE)	$\mathrm{RMSE}=\sqrt{\frac{\sum_{i=1}^{n}{\left(y_{i}-{\overset{\text{˜}}{y}}_{i}\right)}^{2}\;\;}{n}}$
Coefficient of determination (R^2^):	
$R^{2}={\left(\frac{n\sum_{i=1}^{n}y_{i}{\overset{\text{˜}}{y}}_{i}-\sum_{i=1}^{n}y_{i}\sum_{i=1}^{n}{\overset{\text{˜}}{y}}_{i}}{\sqrt{n\sum_{i=1}^{n}y_{i}^{2}-{\left(\sum_{i=1}^{n}y_{i}\right)}^{2}}\sqrt{n\sum_{i=1}^{n}{\overset{\text{˜}}{y}}_{i}^{2}-{\left({\overset{\text{˜}}{y}}_{i}\right)}^{2}}}\right)}^{2}$	
Mean absolute error (MAE):	$\mathrm{MAE}=\frac{\sum_{i=1}^{n}\left|y_{i}-{\overset{\text{˜}}{y}}_{i}\right|}{n}$
Pearson correlation coefficient:	
$r_{\mathrm{xy}}=\frac{n\sum_{i=1}^{n}x_{i}y_{i}-\sum_{i=1}^{n}x_{i}\sum_{i=1}^{n}y_{i}}{\sqrt{n\sum_{i=1}^{n}x_{i}^{2}-{\left(\sum_{i=1}^{n}x_{i}\right)}^{2}}\sqrt{n\sum_{i=1}^{n}y_{i}^{2}-{\left(\sum_{i=1}^{n}y_{i}\right)}^{2}}}$	
